# Peristomal intestinal metaplasia with response to serial electrosurgery

**DOI:** 10.1016/j.jdcr.2024.01.002

**Published:** 2024-01-13

**Authors:** McKayla Poppens, Thomas Hester, Sruthi Renati, Allison C. Billi, Lori Lowe, Julie E. Mervak

**Affiliations:** a David Geffen School of Medicine at University of California Los Angeles, Los Angeles, California; b Department of Dermatology, University of Michigan, Ann Arbor, Michigan; c Department of Pathology, University of Michigan, Ann Arbor, Michigan

**Keywords:** cutaneous intestinal metaplasia, ectopic gut tissue, ileostomy, inflammatory bowel disease, ostomy, peristomal, stoma

## Introduction

Peristomal dermatologic complications are common, including contact dermatitis, infection, maceration, malignant neoplasm, pyoderma gangrenosum, pressure ulcer, or preexisting dermatoses such as psoriasis.^1^ Peristomal intestinal metaplasia (PIM) has been infrequently described. We describe a case of PIM successfully treated with serial electrosurgery at 4-week intervals.

## Case report

A 78-year-old woman with a history of ulcerative colitis, treated with diverting loop ileostomy at 16-years-old, was referred to dermatology for evaluation of 1-year history of peristomal erythema, itching, and mild pain. She noted mild increase in stool output from her stoma around the onset of her symptoms. She denied previous similar peristomal concerns or proceeding trauma. Her ulcerative colitis had been well controlled since surgery, and she was otherwise healthy.

**Fig 1:**
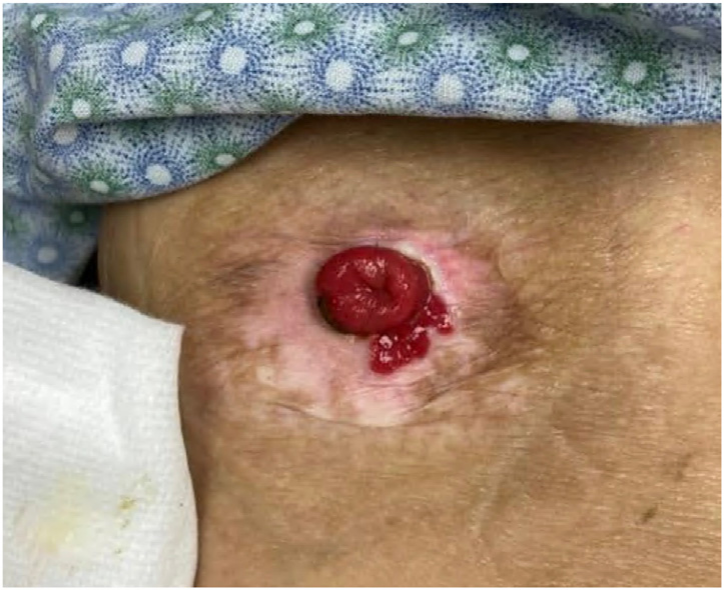
Clinical appearance of the peristomal red eroded thin plaque at initial presentation.

**Fig 2:**
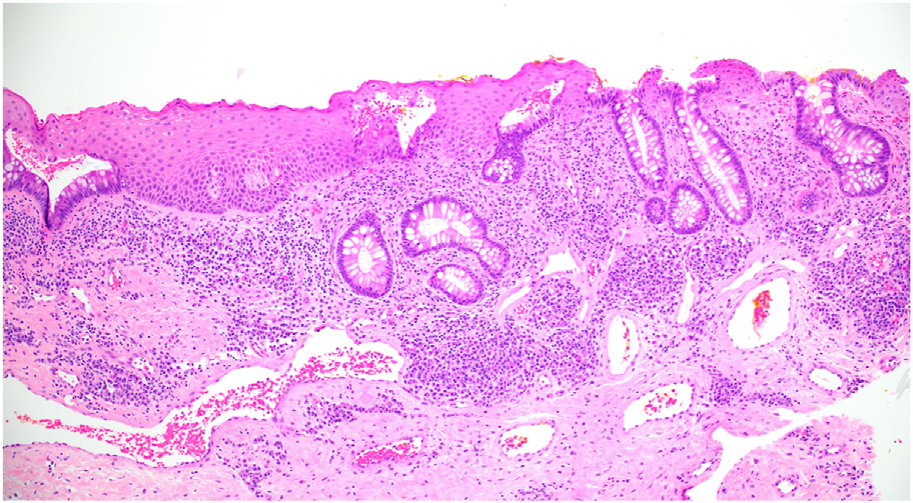
Skin biopsy of peristomal intestinal metaplasia demonstrating mild epidermal hyperplasia with numerous small intestinal colonic crypts that connect to the epidermal surface. In the dermis is a moderate inflammatory infiltrate of lymphocytes and plasma cells. (Hematoxylin-eosin stain; original magnification: × 10.)

**Fig 3:**
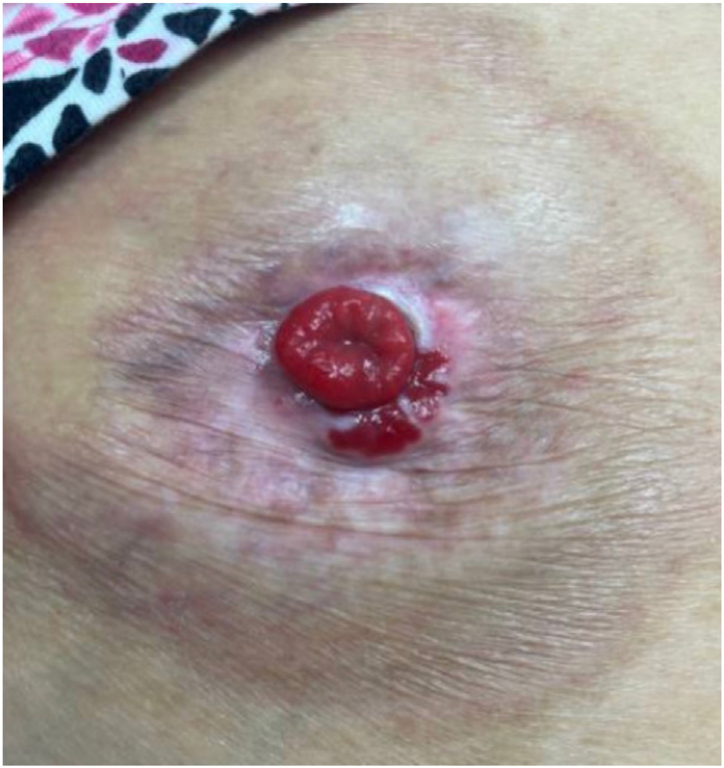
Persistence of peristomal intestinal metaplasia with 3 sessions of electrosurgery at 12-week intervals.

**Fig 4:**
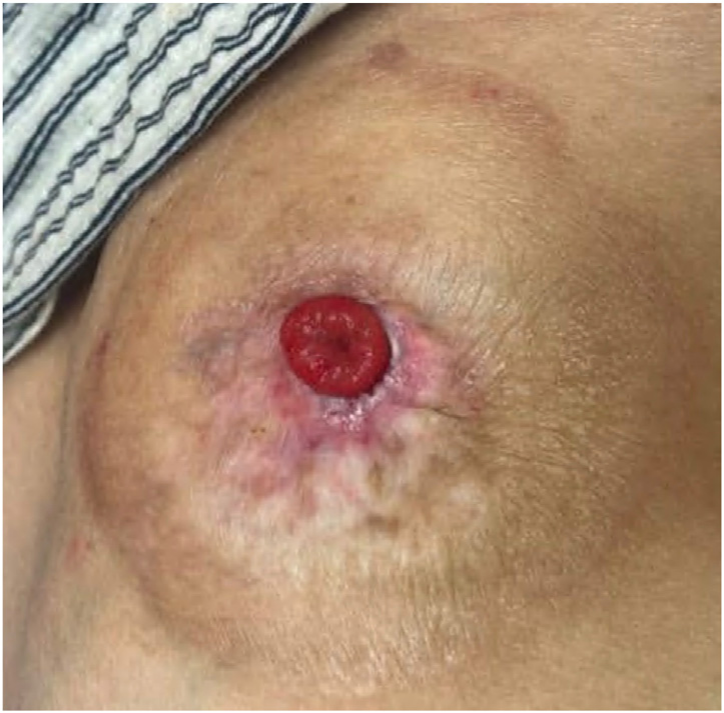
Resolution of peristomal intestinal metaplasia after 3 sessions of electrodessication at 4-week intervals.

Physical examination revealed a well-demarcated, red, superficially eroded plaque with digitate projections at the inferomedial border of the stoma (Fig 1). She was treated for suspected irritant contact dermatitis with clobetasol 0.05% solution over 10 months without improvement. Punch biopsy of the plaque was performed, and histology showed mild epidermal hyperplasia with numerous small intestinal colonic crypts connecting with the epidermal surface and a moderate number of lymphocytes and plasma cells in the dermis (Fig 2). A diagnosis of PIM was made. Serial intralesional triamcinolone (5-10 mg/mL) was performed at 4- to 8-week intervals without improvement. The patient was then treated with serial electrodessication. Three initial sessions were performed at 12-week intervals (local anesthesia with lidocaine hydrochloride 1% with epinephrine 1:100,000, single pass, low-terminal setting, power 4.0 watts) (Fig 3). Given unsatisfactory clinical response, treatment was escalated. The patient received 3 additional sessions (local anesthesia with lidocaine hydrochloride 1% with epinephrine 1:100,000, 2 passes, low-terminal setting, power 8.0 watts) every 4 weeks leading to complete resolution (Fig 4).

## Discussion

Of the 10 previous cases of PIM in the literature, 50% were in patients with inflammatory bowel disease, and of those, 80% had a history of ulcerative colitis.^1^, ^2^, ^3^ Eight previous cases provided data on length of time between stoma placement and onset of PIM with median time to onset of 30 years.^1^^,^^3^, ^4^, ^5^, ^6^ Six previous PIM cases discussed interventions, all of which were physical destructive methods—curettage, electrocautery, surgical excision, or stoma revision.^1^^,^^3^, ^4^, ^5^, ^6^ Silver nitrate application was utilized in one case along with curettage,^4^ but no other topical medications have been reported as effective. The case that used surgical excision reported sustained resolution 2 years after treatment^3^; however, most did not report long-term follow-up.^1^^,^^4^^,^^5^

It has been hypothesized that the proliferation of intestinal tubular glands in peristomal skin occurs secondary to implantation as suture passes through bowel mucosa and skin during ostomy surgery.^3^^,^^4^ However, this does not explain the significant delay in PIM presentation—averaging 30 years in previously reported cases and more than 60 years in our patient—suggesting decreased natural resistance of the skin to PIM with age. An alternative theory is that skin exposure to bile salts in stool promotes intestinal epithelial migration via a mechanism involving NF-κB.^7^ Resolution with destructive methods, such as electrosurgery, supports the hypothesis that destruction of the intestinal tubular glands eliminates secretory factors resulting in PIM.^5^

The diagnosis of PIM should be considered in patients with longstanding ostomies, particularly of several decades, with no previous similar peristomal complications. Punch biopsy should be performed to exclude alternative diagnoses including adenocarcinoma and steroid-responsive conditions such as contact dermatitis. Given the dramatic efficacy of biologic therapies in the management of inflammatory bowel disease, and subsequent shift away from surgical management, the frequency of decades-old ostomies is likely to decline. This is still a diagnosis that dermatologists must consider to prevent delays in care.

In 2 previous case reports of PIM, reepithelialization using electrosurgery was utilized and each required multiple treatments.^1^^,^^5^ Notably, our patient did not demonstrate clinical improvement until treatment frequency was moved to monthly and hyfrecator wattage increased. Based on these collective outcomes, PIM patients should be counseled that multiple treatments, in relatively close succession, are likely necessary for clinical response.

## Conflicts of interest

None disclosed.
